# Diagnostic Utility of Flow Cytometry in Myelodysplastic Syndromes

**DOI:** 10.3389/fonc.2016.00161

**Published:** 2016-06-27

**Authors:** Carmen Mariana Aanei, Tiphanie Picot, Emmanuelle Tavernier, Denis Guyotat, Lydia Campos Catafal

**Affiliations:** ^1^CNRS UMR5239, Université de Lyon, Saint-Etienne, France; ^2^Laboratoire d’Hématologie, CHU de Saint-Etienne, Saint-Etienne, France; ^3^Institut de Cancérologie Lucien Neuwirth, Saint Priest en Jarez, France

**Keywords:** myelodysplastic syndromes, phenotypic aberrancies related to dysplasia, myeloid maturation patterns, FCSS scores, prognostic value of immunophenotypic scores

## Abstract

Myelodysplastic syndromes (MDSs) are clonal disorders of hematopoiesis that exhibit heterogeneous clinical presentation and morphological findings, which complicates diagnosis, especially in early stages. Recently, refined definitions and standards in the diagnosis and treatment of MDS were proposed, but numerous questions remain. Multiparameter flow cytometry (MFC) is a helpful tool for the diagnostic workup of patients with suspected MDS, and various scores using MFC data have been developed. However, none of these methods have achieved the sensitivity that is required for a reassuring diagnosis in the absence of morphological abnormalities. One reason may be that each score evaluates one or two lineages without offering a broad view of the dysplastic process. The combination of two scores (e.g., Ogata and Red Score) improved the sensitivity from 50–60 to 88%, but the positive (PPV) and negative predictive values (NPV) must be improved. There are prominent differences between study groups when these scores are tested. Further research is needed to maximize the sensitivity of flow cytometric analysis in MDS. This review focuses on the application of flow cytometry for MDS diagnosis and discusses the advantages and limitations of different approaches.

## Introduction

The diagnosis and prognosis of myelodysplastic syndromes (MDS) relies on cytology and cytogenetic data, but recent progress in Multiparameter flow cytometry (MFC) improved the sensitivity required to obtain a reassuring MDS diagnosis in the absence of prominent morphological abnormalities. An important drawback of the immunophenotypic method in MDS is related to the absence of the antigen imprints associated with dysplasia. The most commonly used method is the pattern recognition-based approach. However, flow cytometry in MDS immunophenotyping has progressed over the last several years with the development of 4- to 10-color multi-laser cytometers, the development of monoclonal antibodies directed toward an increased number of antigens on hematopoietic cells, the discovery of new fluorochromes, and the development of innovative software for flow cytometry data analysis. The new analysis programs allow better discrimination of the cell populations using the Automatic Population Separator (APS), which provides the largest view of cell antigenic expression using a backbone-based analysis strategy (i.e., the Calculate Data tool from Cytognos Infinicyt™ Flow Cytometry Software, Salamanca, Spain) or the construction of databases, which provide a better understanding of the normal maturation pathways of bone marrow (BM) cells and offer a tool to interpret the different maturation abnormalities in a non-subjective manner.

Data in the field of MDS immunophenotyping remain scarce and contradictory despite this progress, primarily due to the lack of standardization in antibody combination panels, cytometer settings, and analysis strategies.

This review focuses on the application of flow cytometry for MDS diagnosis and discusses the advantages and limitations of different approaches.

## MFC Analysis in MDS

Multiparameter flow cytometry is increasingly used to reinforce MDS diagnosis (particularly for reassuring the diagnosis of the low-grade MDS and “MDS unclassifiable” categories), determine the prognostic outcome, and monitor the evolution of MDS patients during therapeutic interventions. The presence of three or more phenotypic abnormalities involving one or more of the myeloid lineages may be considered “suggestive” of MDS, but flow cytometric abnormalities alone in the absence of conclusive morphological and/or cytogenetic features are not diagnostic of MDS (1).

Multiparameter flow cytometry assesses dysplastic changes in maturing myeloid cell compartments (e.g., neutrophil, monocytic and erythroid lineages) and evaluates immature progenitor compartments (e.g., myeloid and B lymphoid lineages).

Several phenotypic aberrancies related to dysplasia were described for this purpose and included in different scoring systems. The analytic strategies are based on interpretations of surface marker abnormalities (e.g., increased or decreased fluorescence intensity of antigens compared to normal BM counterparts, asynchronous expression of antigens, or lineage-aberrant expression). Several quantitative differences in immature progenitor compartments versus normal counterparts were also noted (e.g., increased percentages of myeloid progenitors and diminished percentages of B lymphoid progenitors).

### Analysis of Immature Progenitor Compartments

Blast quantification is essential for MDS and acute myeloid leukemia (AML) because the blast count contributes to the WHO classification of MDS/AML and is part of all prognostic scoring systems (2, 3). Generally, a good correlation is observed between MFC and morphologic evaluation, but sometimes differences can occur, mainly in cases with higher blast counts in cytology (2). The differences can occur if the MFC samples are hemodiluted (2, 3).

Recently, to overcome this problem, bone marrow purity (BMP) assessment has been proposed. For example, the BM blast percentage obtained in MFC should be adjusted according to the number of lymphocytes. The method has limitations at the extremes of hemodilution, where slight variations in the lymphocyte number result in significant changes in BMP percentage. The cut-off of the BMP was set at 40%; below this value, the MFC analysis significantly underestimates the blast count (3).

The “Ogata score” was the first score developed as a screening test, primarily for the evaluation of immature progenitor compartments (4).

This score includes four parameters: the percentage of CD34^+^ myeloid progenitor cells in the BM, the frequency of B-cell precursors within the CD34^+^ compartment, CD45 expression on myeloid progenitors relative to CD45 expression on lymphocytes, and the evaluation of neutrophil granularity by comparison to SSC on lymphocytes. This score was tested in a prospective validation study comprising 134 low-grade MDS and 106 control BMs enrolled in two centers from Japan and Italy. The diagnostic sensitivities were 65 and 89% and the specificities were 98 and 90% for a score ≥2 for the Japanese and Italian cohorts, respectively. Three other parameters (expression of CD11b, CD15, and CD56) were analyzed on CD34^+^ myeloblasts, but the results demonstrated that these parameters did not improve the diagnostic power (5).

However, the “Ogata score” has limited applicability in hypocellular BM samples and samples from pediatric patients (6).

A recent French multicenter study confirmed the feasibility of the Ogata score for MDS diagnosis, but highlighted its limitations in low-risk MDS (7).

The aberrant expression of lymphoid markers on CD34^+^ myeloblasts was observed in small numbers of MDS cases. The presence of CD5 was found in only 1.8% of MDS patients in an MLL Munich Leukemia Laboratory study (8) and 1.6% of MDS cases enrolled in a French multicenter study (7). The presence of CD7 on myeloid progenitors was observed in 3.5% (Kern study) and 9% of MDS BM samples in a French study (7).

These studies confirm previous Ogata data and reflect no need to include evaluation of CD5, CD11b, or CD15 and little need to include CD56 and CD7 expression in myeloid progenitor compartments in the performing MDS diagnosis.

The fact that the overestimation of CD5, CD7, and CD56 expression may be due to inappropriate comparisons with the background of the blasts was highlighted (9), and this comparison may underlie the reason for the percentage discrepancy between the Ogata and French multicenter studies.

Kussick and coworkers performed a diligent study of more than 800 samples and found that antigens were expressed abnormally by 50% or more of the cases in the MDS patient group, including HLA-DR, CD13, CD33, CD38, and CD117 (10).

There is a well-known physiological age-related variation in the precursor B-cell compartment composition (11). This variation is more evident in very young children below the age of 2 years (12).

In conclusion, the convenient interpretation of immunophenotyping results performed for evaluation of the B-cell precursor compartment requires the use of adequate reference data.

A diminution of B-lymphoid precursors was also observed in many primary immune deficiencies associated with agammaglobulinemia (12).

### Analysis of Mature Neutrophils

The “Ogata score” also includes a criterion for neutrophil lineage evaluation, the morphometric parameter granularity, which is evaluated as a ratio between the side scatter (SSC) of granulocytes and the SSC of lymphocytes (the threshold for normal samples was set at 6). The specificity of the granulocyte/lymphocyte (Gra/Ly) SSC ratio was reported at 89% (7), but discordance with the cytology evaluation was also reported (13). Two possible situations cause these differences: the increased proportion of mature circulating neutrophils due to BM hemodilution, which affects the Gra/Ly SSC ratio (14), and the presence of less than 10% dysplastic granulocytes (13). Flow cytometry seems more informative than cytology in the latter case (i.e., refractory anemia with or without ring sideroblasts, in which dysgranulopoiesis may not exceed 10% of granulocytes according to the WHO recommendations) (13).

The Gra/Ly SSC peak channel ratio improved reproducibility, and it was the most useful for discriminating the differences in hypogranularity between MDS patients and other pathological conditions (14).

Numerous aberrant phenotypes are related to neutrophil dysplastic changes.

Kern et al. developed a screening score that includes five MDS-typical aberrant antigen expression levels in the neutrophil compartment: abnormal expression of CD13/CD16 and CD11b/CD16, aberrant expression of CD56, and lack of CD33 and CD64 expression (15).

**Figure 1:**
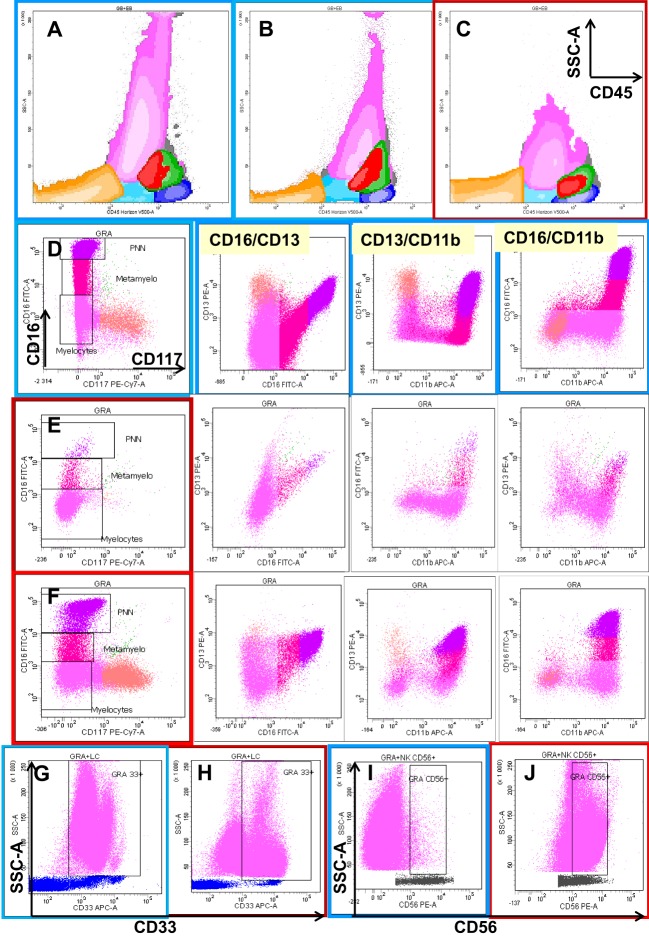
**Eight-color flow cytometric analysis of the different cellular compartments in bone marrow (BM) aspirates with a focus on granulocytic lineage maturation**. The first row shows a global overview of BM cellular compartments projected on SSC/CD45 graphs, including erythroblasts (orange), the CD45^low^ progenitor/precursor compartment (light blue), monocytic lineage precursors (red), more mature monocytic cells (green), and lymphocytes (dark blue). Dot plot **(C)** shows the neutrophil and monocyte hypogranularity compared to two normal cases **(A,B)**. The next three rows show the progression from CD117^+^ CD13^+^ CD16^−^ neutrophil precursors (salmon color) through two intermediary stages of maturation from CD117^−^ CD13^−/+low^ CD16^−^ (deep pink) and CD13^−/+low^ CD16^+var^ (crimson) to mature CD13^+hi^ CD16^+hi^ (fuchsia) neutrophils in one normal bone marrow **(D)** and two MDS cases **(E,F)**. Note the differences in the maturation patterns evaluated on CD16/CD13, CD13/CD11b, and CD16/CD11b plots between the normal BM and MDS cases. The last row shows two types of aberrancies observed in granulocytes in MDS settings compared with normal BM: the absence of CD33 **(H)** compared to normal expression **(G)**, and the aberrant expression of CD56 **(J)** expressed as a 1-log difference compared with normal granulocytes **(I)**.

Figure 1 shows examples of the interpretation of flow cytometric profiles of the mature neutrophil compartment with respect to the analysis of dysplasia as described by Kern and collaborators.

Dyssynchronous expression of CD13/CD16 and CD11b/CD16 was considered if the discordance detected, compared to the “normal” pattern, was at least a half-log of the signal intensity in at least one parameter (8, 13). Possible drawbacks in interpretations of the loss of CD16 are related to the inclusion of eosinophils in the neutrophil gate and the presence of apoptotic cells, which are also observed in two pathological non-MDS conditions, e.g., paroxystic nocturnal hemoglobinuria and patients with a genetic polymorphism (16).

Even the International/European LeukemiaNet (ELN) Working Group for Flow Cytometry in MDS considers the aberrant expression of CD56 on granulocytes as an MDS-associated feature, but the interpretation of this parameter may raise problems of misinterpretation (e.g., aberrant expression should be considered if the CD56 expression is superior to at least a 1-log decade of signal intensity compared to normal controls) (9, 16). Increased CD56 expression is also found on activated granulocytes (9).

Relatively low-level CD56 expression on 10–25% of maturing granulocytes is observed in the setting of BM regeneration with or without granulocyte colony-stimulating factor therapy (17).

An absence of CD33 was rarely reported, and it may be related to a polymorphism. The altered expression of this marker in this case concerns all myeloid populations (e.g., myeloid progenitors, neutrophils, and monocytes) (9).

CD64 absence or diminution is variable, and this variation may be the reason that it was not included in the last ELN recommendation (6).

**Figure 2:**
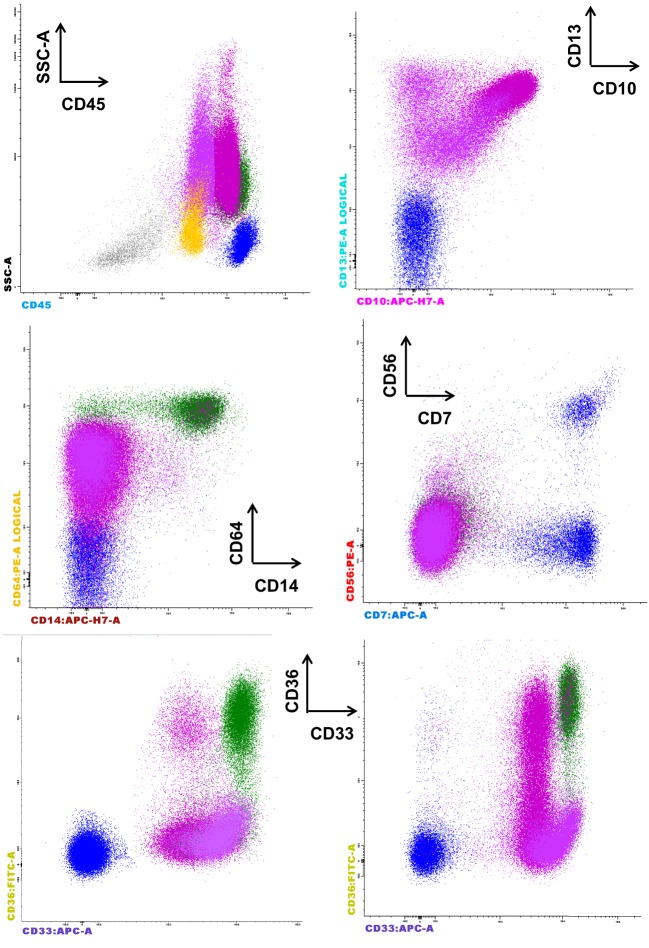
**Representative case of refractory anemia with excess of blasts for which the parameters of the Kern scores are not informative, but the CD36 expression on mature CD10^+^ granulocytes is remarkable (last row, left side) compared to normal counterpart (last row, right side)**. The figure depicts the significant reduction of erythroblasts (gray), the proliferation of CD45^+low^ blasts (yellow), and the aberrant expression of CD36 on CD10^+^ granulocytes. The aberrant expression of CD56 is not observed on monocytes (CD33^+hi^ CD36^+^ CD64^+^ CD14^+^, green color) or granulocytes (CD13^+var^ CD64^+low^ CD33^+^ CD10^−^ immature granulocytes, deep pink, and CD13^+hi^ CD64^+low^ CD33^+int^ CD10^+^ mature granulocytes, fuchsia). The absence of CD33 expression on the granulocytes is not noted.

The European LeukemiaNet Working Group proposed other parameter evaluations for phenotypic dysplastic changes on granulocytes, including the abnormal expression of CD36 and CD10 and an aberrant CD15/CD10 pattern (6). A possible upregulation of CD36 on the granulocyte surface may be related to cell apoptosis, and this marker is required for phagocytosis by macrophages and in the suppression of macrophage proinflammatory functions, consequently acting on the synthesis of proinflammatory cytokines, such as TNFα, IL-1β, and IL-6, as well as inducible nitric oxide synthase (iNOS) (18). Figure 2 shows the abnormal expression of CD36 on mature granulocytes (CD10^+^) in a case of refractory anemia with excess of blasts (RAEB).

### Analysis of Mature Monocytes

**Figure 3:**
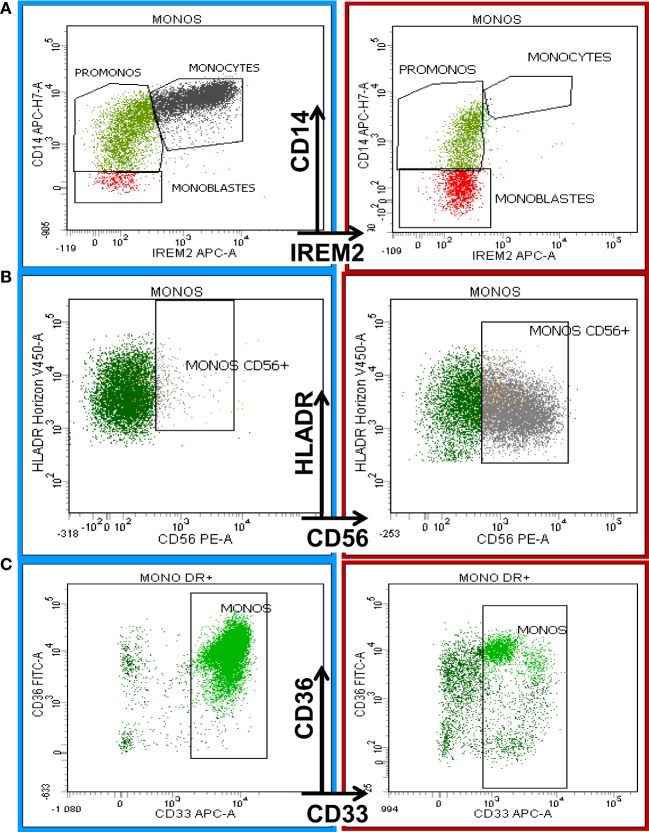
**Eight-color flow cytometric analysis of monocytic antigenic expression in a normal BM sample (left column) compared with a case of chronic myelomonocytic leukemia (CMML, right column)**. The first row shows the maturation profile of the monocytic lineage delineated by CD14 and CD300e (IREM-2) expression according to the Euroflow analysis strategy (19). The right plot in **(A)** shows monocyte maturation arrest. **(B)** shows the aberrant expression of CD56 on monocytes in a CMML patient (right) compared with normal BM (left). **(C)** The last row depicts the diminishing expression of CD33 on mature monocytes in a CMML patient (right) compared to a normal counterpart from a healthy donor (left).

The following most frequent phenotypic changes were observed on mature monocytes: decreased SSC; arrest of maturation (Figure 3A); diminution of CD45, CD11b, HLA-DR, and CD15 expression; aberrant pattern of HLA-DR/CD11b; abnormal expression (higher or lower) of CD16 on CD11b^+^ monocytes; abnormal expression of CD36, CD56 (Figure 3B), and CD7; partial absence of CD13 or CD33 (Figure 3C); and abnormal pattern of CD36/CD14 (6).

### Analysis of the Erythroid Compartment

The European LeukemiaNet Working Group describes four major abnormalities: the increased percentage of immature CD117^+^ erythroid precursors, the abnormal heterogeneous and low expression of CD36 and CD71, and an aberrant pattern of CD71/CD235 expression (6).

A useful tool for the evaluation of erythroid lineage is the Red score, developed by Mathis et al. (20). This score takes into account the following three parameters: the coefficient of variance of CD36 and CD71 on CD36^+^ CD64^−^ CD71^+^ erythroblasts and the hemoglobin value. A score of ≥3 is very useful to detect the dysplastic changes observed on erythroid lineages, with a sensibility of 77.5%, specificity of 90%, positive predictive values (PPV) of 97%, and negative predictive value (NPV) of 49% (20). The authors affirm that a major drawback of the evaluation of dysplastic changes in the erythroid lineage using cytometry is red cell lysis during sample preparation. A possible improvement could be obtained using a nuclear dye (e.g., CyTRAK orange, DRAQ5, or DRAQ7) that allows the discrimination of nucleated and non-nucleated cells in MCF (20).

However, a recent study shows that the lysis method has the most consistent quantitative antigen intensities (21).

The CV evaluation of CD36 and CD71 on CD36^+^ CD64^−^ CD71^+^ erythroblasts has been proposed as most useful for evaluations of dysplastic changes in erythroid lineages (20, 21). Other markers can also be informative for dysplastic changes in the erythroid lineage, such as CD235a (20–26) and CD105 (21).

A recent study conducted to expand the understanding of immunophenotypic analysis of erythropoiesis and further evaluated the dysplastic changes of the erythroid lineage in MDS included the following characteristics: the intensity of expression of CD71, CD36, CD105, CD235a, and CD117; the frequency of CD105^bright^ erythroid cells; and the proliferation index of the immature (CD105^+^) and mature (CD235a^+^, CD71^+^, and CD36^+^) erythroid cells. Changes in the CD36 and CD71 intensity were frequently observed in the MDS cohort, as were a loss of mature erythroid progenitors and an increased frequency of the CD105^bright^ population.

The incorporation of other erythroid antigen aberrancies, such as that of CD105, into flow cytometry scores would allow the detection of low-grade MDS, but prospective studies are necessary (21).

A good correlation between the phenotypic abnormalities of the BM erythroid compartment in MDS patients and the increased percentage of nucleated red blood cells in the circulation that is a good discriminator between regenerative and non-regenerative anemias, making this parameter a good choice for erythroid evaluation scores. The new parameters appear to be sensitive for the detection of small changes in the number of red cells with inadequate hemoglobinization (reticulocyte hemoglobin content – Ret He and the percentage of hypochromic red cells – % Hypo He, which reflect the iron status over the previous 2–3 months). They are also useful for the detection of the circulatory immature reticulocyte fraction (IRF), which has been found to be elevated in MDS settings (27, 28). These parameters, in addition to the automated reticulocyte count, can be used to identify qualitative abnormalities of erythropoiesis.

### Potential Utility of Multiparameter Flow Cytometric Scoring Systems in Myelodysplastic Syndromes

Over time, several flow cytometric scoring systems have been developed in order to screen multiple cell lineages and to provide information that could be useful both for diagnosis and for determining disease prognosis.

### Diagnosis Utility

The first FCSS was developed by Wells and collaborators in 2003, and study was conducted on a cohort of 115 patients with a diagnosis of *de novo* or secondary MDS who subsequently underwent allogeneic hematopoietic stem cell transplantation. The immunophenotypic abnormalities were categorized as normal/mild (0–1), moderate (2–3), or severe (≥4). The Wells FCSS correlated inversely with the leukocyte and absolute neutrophil counts and correlated directly with IPSS scores and with IPSS cytogenetic risk categories (29).

Comparison of high-resolution cytogenetics tests and the phenotypic abnormalities detected using the Wells FCSS showed that the immunophenotypic test was positive in 100% of array-positive MDS specimens and that higher flow cytometric abnormality scores correlated with increasing complexity of genomic abnormalities (30). Moreover, in patients with clonal abnormalities associated with MDS, the Wells FCSS had a good specificity [did not detect phenotypic abnormalities indicating myelodysplasia in 68 of 79 CGH-negative specimens (specificity of 86%)] and a good sensitivity [the immunophenotypic abnormalities suggestive of MDS were identified in 18 of 20 CGH-positive specimens (sensitivity of 90%)] (31). Recent studies state that quantifying immunophenotypic aberrancies by FCSS is useful in MDS diagnosis, especially for identifying patients with a high likelihood of having MDS among patients with unexplained cytopenias (17, 32). However, the revised guidelines for the integration of flow cytometry results in the WHO classification of MDS, a proposal of the International/European LeukemiaNet (I/ELN) Working Group for Flow Cytometry in MDS, recommend that further studies are necessary to establish whether in patients with unilineage dysplasia, normal karyotype, and no detected mutations, the presence of aberrant immunophenotypes provides added value in the diagnostic workup (6). The conclusion of the I/ELN Working Group was that no definitive diagnosis should be given if the MCF report is not integrated with the other diagnostic information provided by clinical information, blood and BM cytomorphology interpretation, cytogenetics, and molecular genetics (6). This reflects the limited value of actual MFC assessment in MDS diagnosis.

### Prognostic Utility

In 2007, a consensus was reached for cases where multiple phenotypic abnormalities are found in MCF. Multiple abnormalities should be regarded as indicative of clonal myeloid malignancy but do not have the prognostic value of immunophenotypic scores. The likelihood of a myeloid neoplasm increases with the number of phenotypic deviations. In the diagnostic work-up in suspected MDS, flow cytometry is of value in the quantitative and qualitative assessment of CD34^+^ progenitor cells (blasts), maturing myeloid cells, and monocytes. The results from quantitative assessments may be of particular value when BM smears are of suboptimal quality or missing, or when monocytic cells are extremely immature (CMML versus AML) (33).

The recent report of the I/ELN Working Group showed that higher numbers of immunophenotypic aberrancies correlate with an increased risk of progression in MDS (6).

However, no prognostic value has been described in cases with low FCSS scores, such as patients with refractory cytopenia with unilineage dysplasia (RCUD), refractory anemia with ringed sideroblasts, unclassified myelodysplastic syndromes, and in patients with refractory cytopenia with multilineage dysplasia (RCMD) and low IPSS risk scores.

Recently, a prognostic score has been proposed that includes three parameters: sideward light scatter, CD117 expression of myeloid progenitor cells, and CD13 expression on monocytes. The MFC can refine prognostication within the IPSS-R low-risk category by identifying patients with worse overall survival in cases of high FCSS scores (34).

### Treatment Response Assessment

Low FCSS scores at diagnosis and a decrease in the FCSS during treatment among patients classified within Int-2 and high-risk MDS patients identified those who are likely to respond to treatment with azacitidine (35).

In addition, the aberrant phenotype of myeloblasts (e.g., in particular, the expression of CD7 or CD56 and loss of CD45 or myeloid antigens) seems to have discriminatory value because it identifies non-responders to growth factor therapies, such as erythropoiesis-stimulating agents and granulocyte colony-stimulating factors, among low- and intermediary-risk MDS patients with the greatest response probability according to conventional criteria (e.g., predictive model of Hellström-Lindberg et al.). Moreover, aberrations in myeloblasts acted as significant biomarkers for MDS treatment failure in multivariate analysis (36, 37). However, the preliminary results of this pilot study have to be validated in a larger cohort.

In determining the prognostic outcome in MDS patients who underwent allogeneic hematopoietic stem cell transplantation, the Wells FCSS score has proved to be useful, and the flow cytometric scores correlated with post-transplantation outcome (29).

## Concluding Remarks

Although increasing evidence exists for the utility of MFC in the diagnostic evaluation of unexplained cytopenia and monocytosis, the predictive value of MFC for MDS still needs improvement despite extensive efforts to improve the methodology.

Possible differences may be assigned to antibody clones for staining, antibody combinations, selection of fluorochromes, antibody stability, and analysis strategies.

We suggest that a rating of the different markers used in MDS panels must be evaluated in large multicenter studies.

In addition, the criteria for BM hemodilution evaluation need to be established and validated in multicenter studies in order to set cut-off values needed to ensure the quality of assessment.

Likewise, it may be necessary to define non-qualifying immunophenotypic markers that could be excluded from different screening scores by extrapolating to non-MDS-qualifying anomalies detected by karyotype test [e.g., isolated loss of the Y chromosome, trisomy 8 (which is frequently associated with aplastic anemia and a very good response to immunosuppressive therapy) and del(20q), which is not associated with clear evidence of MDS (38)] or to other cytopenias of different origins (malnutrition, hematinic deficiency, multisystem organ failure, cytopenia after toxic injury, immune disorders, etc.). New immunophenotypic footprints should be tested.

Immunophenotypic scores must also be developed to predict the treatment response and assess and predict the evolution of so-called clonal cytopenias of undetermined significance (CCUS), which more closely resemble MDS patients than age-matched controls with somatic mutations (39).

In conclusion, morphology should be supplemented with additional new techniques for MDS diagnosis, and the utility of MFC has been demonstrated in the detection of early stages of myelodysplasia when a broad range of myeloid and non-myeloid antigens are evaluated. However, multicenter studies still need to be conducted in order to determine which markers are more informative in discriminating MDS from cytopenias associated with other non-neoplastic causes and for advancing knowledge about the myeloid maturation patterns. Thereafter, it will be necessary to construct robust and practical FCSS that can be implemented in routine procedures.

## Author Contributions

All authors listed, have made substantial, direct, and intellectual contribution to the work, and approved it for publication.

## Conflict of Interest Statement

The authors declare that the research was conducted in the absence of any commercial or financial relationships that could be construed as a potential conflict of interest.
